# Supplementary figures and images for: Motion compensated whole-heart coronary cardiovascular magnetic resonance angiography using focused navigation (fNAV)
Source: J Cardiovasc Magn Reson. 2021 Mar 29;23:33. doi: 10.1186/s12968-021-00717-4 (PMC8006382; doi:10.1186/s12968-021-00717-4)

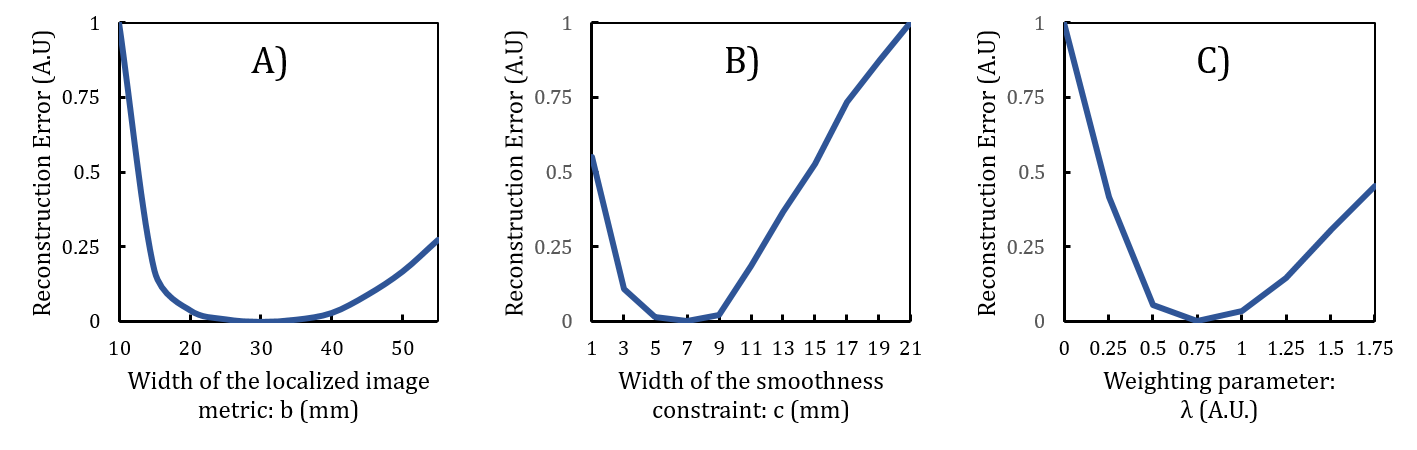

Supplement: Supplementary file 1 — Additional file 1: Figure S1. Optimization of fNAV tunning parameters. Using our numerical simulation with the ground truth images as a reference, the root-mean-square error was calculated for fNAV reconstructions as a function of A) the width of the localized image metric, B) the width of the smoothness constraint, C) the weighting parameter λ. The parameter values (b = 30 mm, c = 7mm, λ = 0.75) that minimized the error was used for all subsequent reconstructions. [file 12968_2021_717_MOESM1_ESM.png]

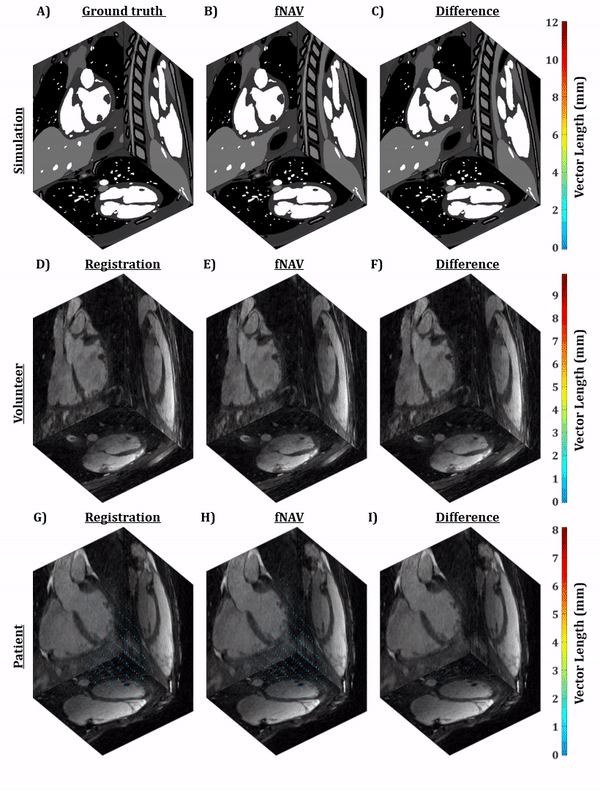

Supplement: Supplementary file 2 — Additional file 2: Video S1. Displacement fields derived from ground truth (A) and fNAV reconstructions of simulated data (B) and the absolute difference (C) overlayed on the ground truth respiratory resolved images. Similarly, displacement fields derived from registering the frames of XD-GRASP reconstructions (D, G) fNAV (E, H) and the absolute difference (F, I) from a healthy volunteer and patient respectively overlayed on the respiratory resolved XD-GRASP reconstructions. [file 12968_2021_717_MOESM2_ESM.gif]
